# Liverwort diversity in China: spatial and taxonomic patterns on species richness

**DOI:** 10.3389/fpls.2024.1513221

**Published:** 2024-12-19

**Authors:** Jiaqi Cui, Xiuhua Yang, Xiaoyu Li, Jitong Li, Siqi Dong, Hongfeng Wang, Chengjun Yang

**Affiliations:** ^1^ Collage of Forestry, Northeast Forestry University, Harbin, China; ^2^ Northeast Asia Biodiversity Research Center, Northeast Forestry University, Harbin, China

**Keywords:** provincial distribution, liverwort species richness, diversity pattern, dominant group, endemic species

## Abstract

The diversity of liverworts in China is rich. It is of great significance to study the species distribution pattern of liverworts in China for the protection of liverworts diversity, flora research and biodiversity monitoring. On the basis of records from national and provincial liverwort lists, herbaria and online databases, a dataset of liverwort distributions was created to analyze the geographical distribution patterns of liverwort species diversity in China. According to the taxonomy of liverwort species in the CoLChina database, more than 60,000 distribution records of 34 provincial geographic units were standardized. ArcGIS 10.8 was used to map the overall richness of liverwort species, as were the individual maps of 14 taxonomic groups of liverworts. Southwest China presented very high species richness, followed by Central China and South China, which presented relatively high species richness. The centers of liverwort species diversity in China are highly consistent with the diversity centers of endemic liverwort in China, many sites from coastal areas to Mountains. The specific distribution centers include the Yunnan-Guizhou Plateau, Hengduan Mountain Range, coastal areas in southern China and Taiwan Mountain Range, as well as the Qinling Mountains and Taihang Mountains in the central region and the Changbai Mountains and Xiaoxing’an Mountains in northeast China. There was significant difference in the distribution patterns of liverwort groups among the different provincial regions in China. As large groups, Jungermanniales and Porellales are absolutely dominant, their distribution patterns are similar to the overall richness of liverwort. The distribution center of Jungermanniales is punctate, while the distribution center of Porellales is flaky. However, the diversity centers of the small and medium liverwort groups are abundant, and their distribution patterns are also significantly different, such as, Marchantiales and Metzgeriales are medium-sized taxa. There were five types of small groups. This study will help us record and understand the biogeographic patterns of liverwort, clarify the geographical distribution of the major phylogenetic groups (order) of liverwort, and analyze the geographical distribution of national endemic and provincial endemic liverworts in China, providing a theoretical basis for future assessments of conservation gaps and reasonable conservation actions for liverworts.

## 1 Introduction

Species richness are a measure of biodiversity, and research conservation and exploitation of biodiversity should begin with their understanding (Lin et al., 2011; Perrino et al., 2023). Species cataloging is the database for biodiversity research, and the data quality and availability of newly collected data are essential for scientific research and conservation planning (Aung et al., 2023). China is one of the countries with the most abundant species of liverwort in the world. Systematic research results on the diversity of liverwort in China have primarily included the Genus Muscorum Sinicorum, which was first published in the 1970s, and more than 600 species of liverworts belonging to 24 families and 106 genera in China have been collected (Chen et al., 1963). The annotated catalog of Chinese Hepaticae and Anthocerotae included 884 species of 52 families and 147 genera of Chinese liverworts and hornworts, which were published in the 1990s (Piippo, 1990). The genera Hepaticopsida et Anthocerotopsida Sinicorum include 923 species of liverworts belonging to 55 families and 156 genera (Gao and Wu, 2010). Based on the Species Catalogue of China (Volume 1 Plants Bryophytes) 1050 species of liverworts belonging to 60 families and 152 genera (Jia and He, 2013). In recent years, the bryophyte part has gradually improved. According to the latest data released of the Catalogue of Life China in 2024, 1191 species of liverworts belonging to 63 families and 176 genera have been identified (Ma, 2006).

In 2002, the Global Plant Conservation Strategy set many goals, among which the current and ongoing loss of plant diversity to slow the pace of global plant extinction (von Konrat et al., 2010). In 2008, within the framework of the Global Plant Protection Strategy, China issued the China Plant Protection Strategy (2010-2020), which formulated an implementation plan for the 16 goals of plant protection in China. The first goal is to establish a species inventory of all Chinese native plants, including bryophytes. However, identifying all relevant species is only one step in protecting China’s liverwort diversity, of which goal five is to ensure the conservation of key areas of plant diversity (Compilation Committee of Plant Protection Strategy of China, 2008). In 2019, the Chinese Plant Protection Strategy (2021-2030) continued to put forward the goal of “promoting the continuous improvement and continuous update of the wild plant protection list” (China Wild Plant Conservation Association (2019). https://www.wpca.org.cn.). The 2021 “Opinions on Further Biodiversity Conservation” also clearly indicate that the overall goal is to continue to promote background surveys and assessments of biodiversity conservation priority areas and national strategic areas by 2025 (XinHua News Agency (2019). https://www.gov.cn/). There are currently documented patterns of species richness in bryophytes in some areas. In 2008, Von Konrat et al. used the flora lists of 400 regions in the world to map the species richness of moss and carried out a study on the world geographic region of bryophytes (von Konrat et al., 2008). The World Moss dataset created by Geffert et al. in 2013 analyzed the global pattern of moss species diversity (Geffert et al., 2013). Qian and Chen updated the bryophyte species richness of each province in China in 2016 and analyzed the relationships between bryophyte species richness and environmental variables (Qian and Chen, 2016). Some botanist conducted a regional study on bryophyte species richness in China in 2021 and analyzed the influence of the contemporary environment and macroevolution on their distribution pattern (Song et al., 2021). However, there are relatively few regional studies on liverwort cretaing a gap in the time scale. In addition, the reliability, regional sampling intensity, and catalog integrity of existing data on liverworts still need to be evaluated. Actual analyses at some continental or global scales are still scarce, and ongoing exploration of existing geographic models is needed to verify them.

In recent years, many changes have taken place in the classification and diversity cataloging of liverworts in China. New taxa are constantly being discovered and described (see **Appendix**), as *Gaolejeunea*, *Sinomylia*, *Soella*, and *Asterellopsis* that were all established on the basis of liverwort species (Zhu et al., 2022). After the discovery of *Marchantia longii*, an endemic species of Yunnan, *Sphaerocarpos siguniangensis* R. L. Zhu & You L. Xiang was discovered on Siguniang Mountain, Sichuan Province, adding a new order of liverworts (Xiang et al., 2016; Xiang and Zhu, 2019). New records of distribution have been reported. Through in-depth investigations and research, domestic and foreign scholars have reported the distributions of many new records of liverwort species in China, such as *Riccardia vitrea* Furuki, located in China and North America (Yin et al., 2021). In the last two years, at least four new records of liverwort have been published every year, and the number of new records published in some regions and taxa is increasing. Some taxa species were excluded, as Chinese scholars found that previous reports of *Porella longifolia* (Steph.) S. Hatt. and *Porella densifolia* var. *robusta* in China were based on errors and were excluded from the Chinese liverwort flora because evidence that the samples could be linked to other species (Qian et al., 2018). These data are widely distributed throughout the relevant literature but are generally available, superfluous and cumbersome, therefore, solid taxonomic knowledge is needed for scientific processing. Accordingly, this study used records of national and provincial liverwort lists, recently published journal articles and an online database of the herbarium to sort out the provincial distribution dataset of liverwort and used quantitative macroecological methods to evaluate the species diversity patterns of liverwort, with the aim of providing the latest data sources for the research and conservation of liverwort resources.

## 2 Data and methods

### 2.1 Data

From the Catalogue of Life China (CoLChina, http://www.sp2000.org.cn/) online database, the liverwort plant list (at the provincial and national levels), the distribution data of liverworts papers published before July 2024 (a total of 250, **Supplementary Data**) and online specimen data (National Specimen Information Infrastructure NSII, http://www.nsii.org.cn/, the Global Biodiversity Information Facility GBIF, https://www.gbif.org/.cn/, Tropicos, Missouri Botanical Garden, https://www.tropicos.org/) were reviewed to compile the distribution of liverwort plant data (**Supplementary Table S1**). For liverwort names obtained from provincial lists, online herbaria and related journal papers, due to typing errors, abnormal authoritative abbreviations and redundant or missing hyphens, manual correction was performed via TNRS (proofreading of names only, not using their species point of view). After obtaining a definitive species name, automatic matching was performed via the ColChina database as a taxonomic reference list. A total of 117 species could not be matched to the ColChina list, so data such as Tropicos, WFO, recently published papers and expert opinions are consulted in turn to unify taxonomic views and determine the correct names. A total of 1305 species of liverwort were included in 63 families and 176 genera in this study. The endemic species were derived from the Species Catalog of China (Volume 1 Plants Bryophytes) and the Genera Hepaticopsida et Anthocerotopsida Sinicorum. The distribution information of the listed endemic liverworts was checked, and their names were revised to determine the endemic status in China.

The distribution information of liverworts was extracted from the literature and specimen data, provincial administrative regions were used as the basic units of data, and standardized processing, verification and screening of the collected liverwort distribution data were conducted. Doubtful specimen species identification, missing collection sites and incorrect records outside of administrative regions in China were eliminated. However, due to the information of “no distribution” is more difficult to determine than that of “distribution”, it is not easy to exclude the distribution information of species included in relevant important works, which may lead to an excessive number of liverwort species in each region. For some species, specimens cannot be found at the present time, however, credible data confirm their distribution in China, and determine their provincial distribution as much as possible based on the source literature.

### 2.2 Methods

The species distribution data collected above are taken as the basis, and 34 provincial-level administrative regions of China are taken as the basic unit of spatial data. To eliminate the influence of area on the species richness assessment, area-related species richness was calculated, i.e., the number of species in a province divided by the logarithmic conversion area of the province (hereinafter referred to as “species richness”) (Yin et al., 2021). The natural break point method was subsequently used to grade the species richness of the liverworts. ArcGIS 10.8 version was used to create a map of all species diversity in the dataset, and different color shadows were used in the map to visualize the recorded species richness levels.

Based on the collected species list, not only can the liverwort species diversity be analyzed, but any subset of the data can also be mapped equivalently. On the basis of the taxonomic view of bryophytes in CoLChina, each species is associated with one of the major liverwort lineages (orders). The liverwort subgroups were plotted by showing the number of species in each region of each group. Finally, the composition of the liverwort floristic boundary was assessed by aggregating data on liverwort species orders and plotting the numbers.

## 3 Results

### 3.1 Species richness pattern of liverwort in China

**Figure 1:**
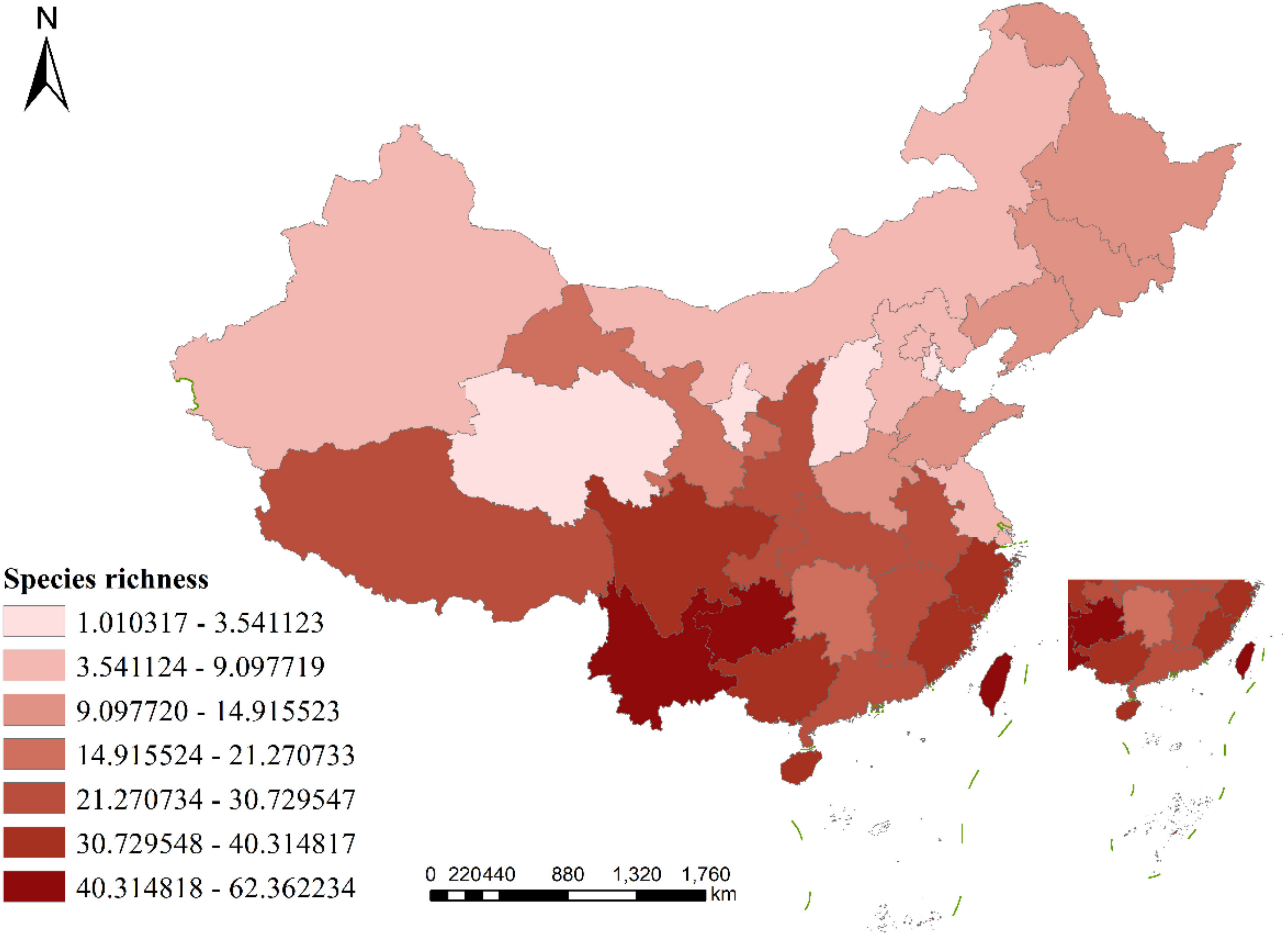
Geographic pattern of species richness of liverworts in China. (Species richness = number of species/ln area).

The distribution of liverwort species has been recorded in all regions of China, but species richness is unevenly distributed among provinces (**Figure 1**). The highest species richness is found in Southwest China, followed by South China and East China, which extend northward to Northeast China and Northwest China, whereas North China and Central China have the lowest species richness. In terms of the distribution of physical geographical areas, the subtropical, tropical and plateau climates are the most concentrated areas of liverwort in each climatic zone. From the perspective of vegetation distribution, the richness of liverwort species is greater in tropical monsoon rainforests and other rainforests, subtropical evergreen broad-leaved forests and temperate mixed coniferous and deciduous broad-leaved forests.

In terms of geographical distribution patterns, the richness of liverwort species in China is high in southern China and low in northern China, and the species distribution varies greatly across provincial regions. The species richness of liverwort in China can be divided into seven gradients: Echelon I, whose species richness ranges from 40.314818-62.362234, has only three province, Yunnan (62.362234), Guizhou Province (52.337595), Taiwan Province (49.263980); Echelon II, whose species richness ranges from 30.729548-40.314817, has five provinces, including and Guangxi Zhuang Autonomous Region (40.314817), Hainan Province (38.693772), Zhejiang Province (37.635506). Echelon III, the species richness ranged from 21.270734-30.729547, with the eight provinces including. Tibet Autonomous Region (30.729547), Jiangxi Province (27.960781), Hubei Province (24.197158), Hong Kong Special Administrative Region (23.803088). Echelon IV, the species richness ranged from 14.915524-21.270733, with two provinces being the Hunan Province (21.270733) and Gansu Province (17.342866). Echelon V, whose species richness ranged from 9.097720-14.915523, had six provinces, including Jilin Province (14.915523), Heilongjiang Province (13.312232), Liaoning Province (12.832078), and Henan Province (11.301309). Echelon VI, whose species richness ranged from 3.541124-9.097719, had six provinces, including the Inner Mongolia Autonomous Region (9.097719), Hebei Province (7.186518), Xinjiang Uygur Autonomous Region (7.059415). Echelon VII, the species richness ranged from 1.010317-3.541123, and the four provinces included Shanxi Province (3.541123), Qinghai Province (3.172555), and Ningxia Province (1.591017). Although some provincial administrative regions had relatively few species, resulting in low calculated species richness, which may reflect only the weakness of field investigations and lack of data collection, the above ranking also reflects the general distribution of the species richness of liverwort in China to a certain extent.

### 3.2 Distribution and diversity patterns of major subgroups

**Figure 2:**
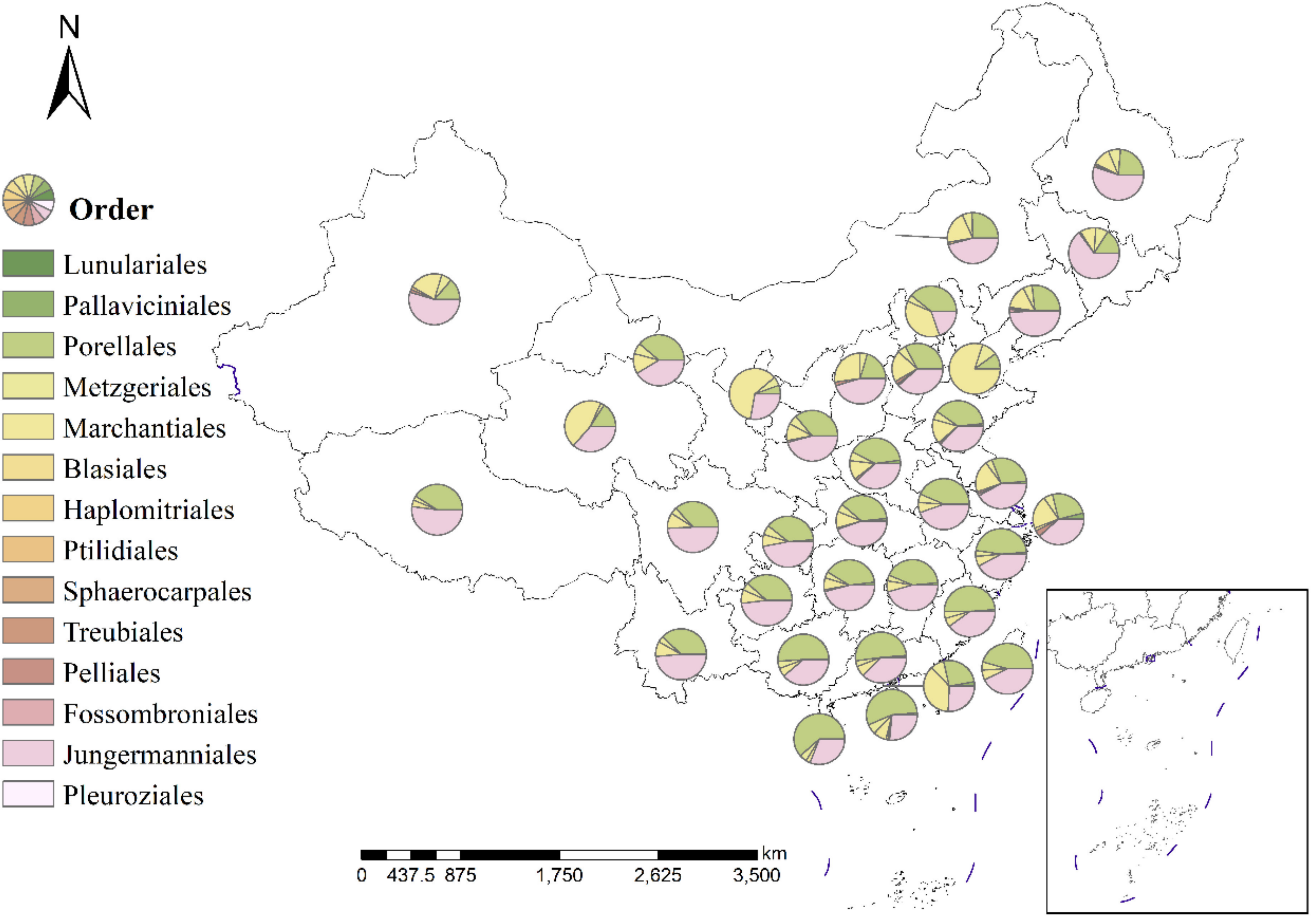
Major orders of liverworts for the main floristic kingdoms in China. The size of the pie charts indicates the total species richness for different orders of liverworts in the respective region.

**Figure 3:**
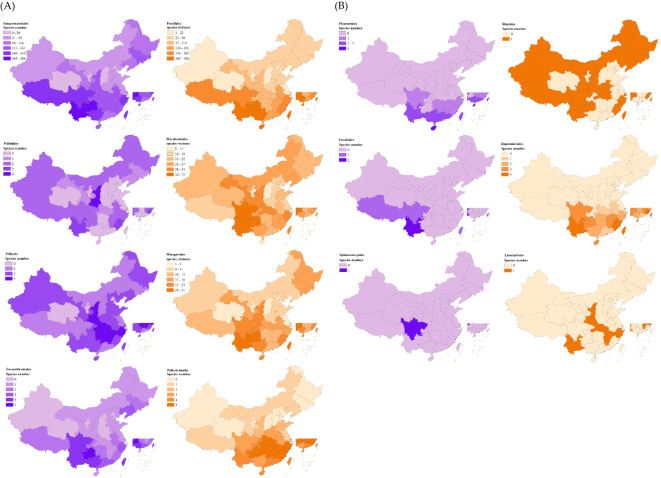
Patterns of species richness for the major subgroups of liverworts in China. Species numbers as species per region. Classification based on COLChina.

The geographical distribution of 14 major liverwort groups (**Figure 2**) revealed that Jungermanniales and Porellales dominated the composition of liverwort species in various provinces of China, but their dominant regions presented obvious differences. Jungermanniales was predominant in 19 provinces, including Southwest China, Northwest China, Northeast China and parts of the middle and lower reaches of the Yangtze River, whereas Porellales was concentrated in 11 provinces, including the southern coastal areas and the middle and lower reaches of the Yellow River. The dominant groups in Tianjin, Macau, Qinghai and Ningxia are Marchantiales and Marchantiaceae, and the liverwort species in this area are widely distributed. The individual subpopulations of the major subpopulations exhibited extremely variable patterns of distribution and diversity (**Figure 3**). The macroorder are defined as orders with more than 500 species, such as Jungermanniales and Porellales (537-573 species). The overall pattern is similar to the total richness of the liverwort, with species distributions in almost every region, indicating certain similarity in species distribution patterns. It mainly refers to the southwest and southern coastal areas of China as the center of species diversity of liverworts in China, and also the center of diversity of macroorder of liverworts, showing a high similarity in the overall distribution area. However, Jungermanniales and Porellales have obvious preference in specific species distribution patterns. In the species distribution pattern of Jungermanniales, the species richness in the southern and northeastern regions is higher than that in the North China Plain and the Loess Plateau. The Yunnan-Guizhou Plateau region showed the highest species richness, followed by the Sichuan Basin-Hengduan Mountains-Southern Himalayas region, the southeastern hilly region-Southern Taiwan Mountains, and the coastal Jiangsu and Zhejiang regions. Porellales show a high species richness in the tropics of China, which is significantly higher than that in the northern region, and the overall diversity distribution center is continuous. In particular, it refers to the southwest of China and the south of the Nanling Mountains. At the same time, the species in the Qinling Mountains, Changbai Mountains and Xiaoxing ‘an Mountains are also more diverse. However, it is worth noting that the geographical distribution of each family in terms of macroorder is different. The species diversity centers of some families are highly similar, such as Frullaniaceae, Lejeuneaceae and Herbertaceae, while some families such as Porellaceae, Anastrophyllaceae and Antheliaceae additionally increase the diversity of the species geographical distribution of this subgroup.

The mesotaxa oders are defined as orders with between 50 and 120 species, such as Marchantiales and Metzgeriales (58-103 species), which are distributed throughout all regions of China. Mesotaxa oders are contained the several genera, such as *Marchantia*, *Reboulia*, and *Metzgeria*. These genera are widely distributed groups. In particular, *Metzgeria conjugata* Lindb., *Metzgeria furcata* (L.) Corda., *Marchantia polymorpha* L., and *Reboulia hemisphaerica* (L.) Raddi of the genera are widely distributed species worldwide. Compared with macroorders, except that the center of species diversity of liverwort is consistent, Marchantiales also has relatively high values in the Tianshan Mountains, Qinling Mountains, Taihang Mountains and southern Changbai Mountains. Metzgeriales presented relatively high species richness in the eastern coastal areas of the Wuyi Mountains, the Qinling Mountains, the northern part of the central Changbai Mountains and the Xiaoxing’an Mountains.

The remaining 10 orders are small orders (1-6 species), including Blasiales, Fossombroniales and Haplomitriales. Most of the orders are not differentiated to a high degree and belong to only a single family and a single genus, but there are great differences in species distribution patterns. Haplomitriales, Sphaerocarpales, Pleuroziales, Lunulariales and Treubiales can be considered the main tropical groups. Although the number of species in these five taxa is not high, almost all of them are distributed in tropical areas of China, such as the Yunnan-Guizhou area, Jiang-Zhejiang area and southeastern hills. *Sphaerocarpos siguniangensis* R. L. Zhu & You L. Xiang is the only species of Sphaerocarpales in China. There are only one or two species in the northern Qinling Mountains extending into the temperate zone and the southern Himalaya in the plateau climate zone, such as *Lunularia cruciata* (L.), Dumor.ex Lindb., and *Apotreubia nana* (S. Hatt. & Inoue) S. Hatt. & Mizut. Among them, Lunulariales is more unique, with a separate tropical-temperate distribution and discontinuous distribution across the whole geographical region. Although Fossombroniales and Pallaviciniales are a small group, the distribution pattern is similar to that of the macroorder and mesotype. The distribution center of Fossombroniales is relatively scattered, mainly concentrated in the southwest, some coastal areas and the south of Changbai Mountain in Northeast China. The distribution center of Pallaviciniales is mainly concentrated in the south of the Yangtze River. The species diversity center of Ptilidiales is Shaanxi Province in the middle reaches of the Yellow River, followed by Yunnan Province and Fujian Province, which have relatively high species richness. The species diversity center of Pelliales was relatively high in Shaanxi Province, Hunan Province, Hubei Province, Jiangxi Province and Zhejiang Province in central China. There is only one species of Blasiales, which has no obvious species distribution center. The geographical distribution areas are patch-like links with discontinuities.

### 3.3 Distribution and diversity patterns of endemic species

**Figure 4:**
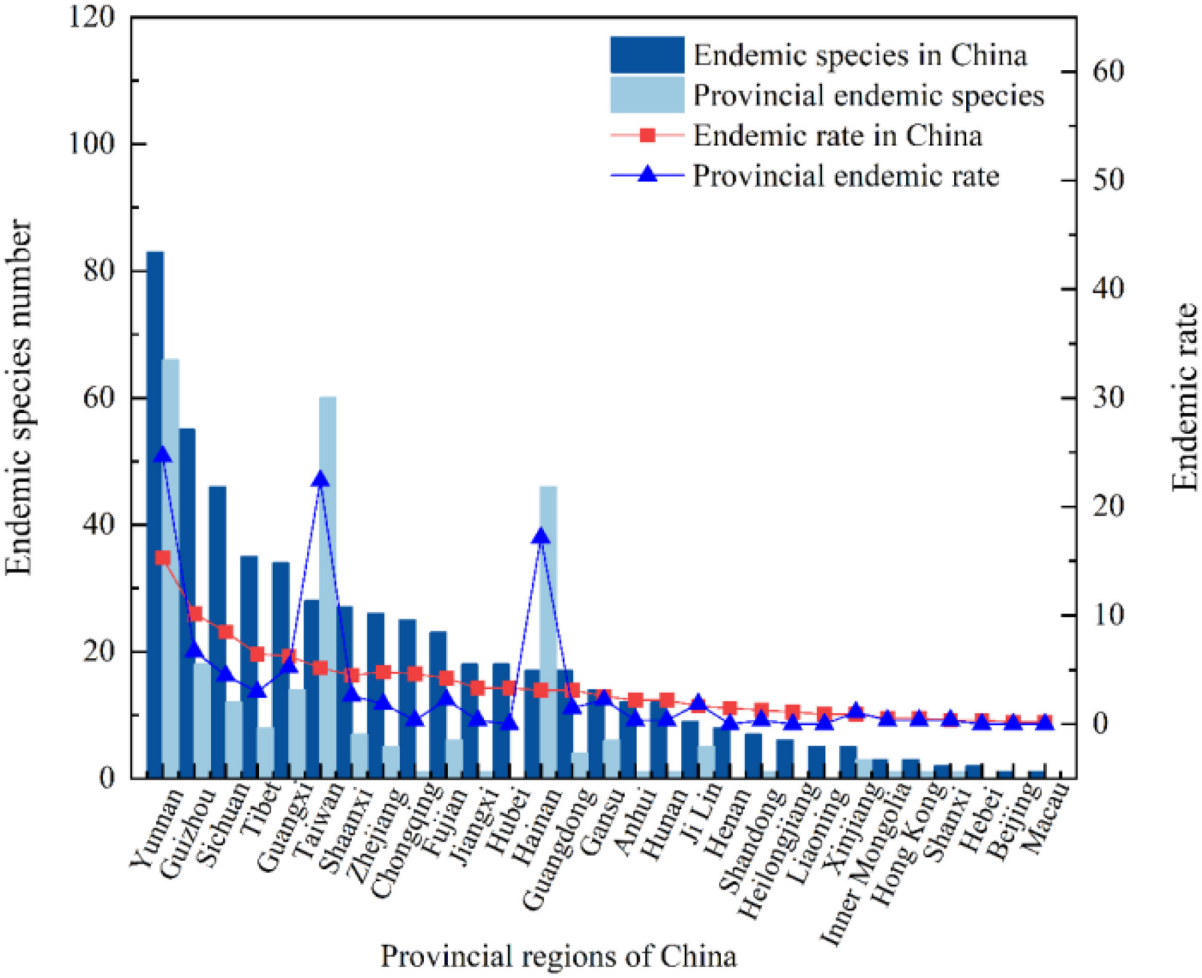
Distribution of endemic species and provincial endemic species in China.

A total of 155 species of endemic liverwort from 28 families and 42 genera were included in this dataset, accounting for 44.44%, 23.86% and 11.88% of the liverwort families, genera and species, respectively in China (**Figure 4**), 29 provinces recorded the distribution of these endemic species. The proportion of endemic species relative to the total endemic species was defined as the endemic rate. The endemic rate in the top 10 provinces with the largest distribution of endemic species ranged from 13.84-53.55. These regions included Yunnan Province (83 species), Guizhou Province (55 species), Sichuan Province (46 species), the Tibet Autonomous Region (35 species), the Guangxi Zhuang Autonomous Region (34 species), Taiwan Province (28 species), Shaanxi Province (27 species), Zhejiang Province (26 species), Chongqing city (25 species), and Fujian Province (23 species). Moreover, the species-level composition of the endemic species of liverwort was analyzed, and the endemic rate in China was not evenly distributed among the families. The endemic rates of most families ranged from 0.65 to 1.94, with a total of 18 families. The endemic rates of some species ranged from 3.32 to 8.39, totaling 8 families. A few cortetes have rates of 10.97-21.29, and only 3 families belong to Frullaniaceae (33 species), Lejeuneaceae (24 species) and Plagiochilaceae (17 species). The geographical distribution of families with high endemic rates may indicate that China is the center for species diversity of these families.

**Figure 5:**
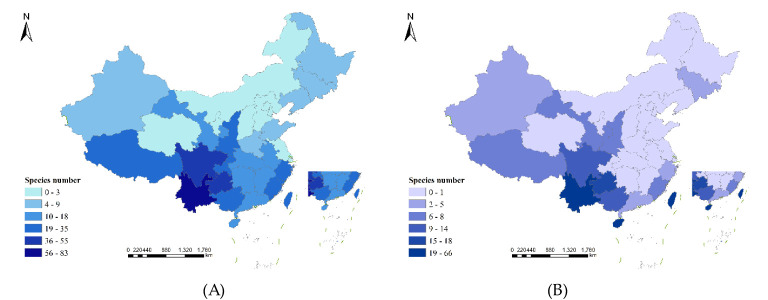
Patterns of species richness for the endemic of liverworts in China. **(A)** Patterns of species richness for the endemic liverworts in China; **(B)** Patterns of species richness for the provincial endemic liverworts in China. Species numbers as species per region.

The provincial endemic species in China are narrowly regional and refer to the liverwort that is confined to a certain province. There are 268 species of liverwort belonging to 37 families and 90 genera in 22 provinces, accounting for 58.73%, 51.14% and 20.54% of the families, genera and species of liverwort, respectively. There are 3 regions in China with more than 40 endemic species, namely, Yunnan Province (66 species), Taiwan Province (60 species) and Hainan Province (46 species), accounting for 64.18% of the provincial endemic species. There are 3 regions with 11–20 provincial endemic species, namely, Guizhou (18 species), Guangxi (14 species) and Sichuan (12 species), accounting for 16.42% of the provincial endemic species. There were 14 regions with fewer than 10 species, accounting for 19.40% of the provincial endemic species (**Figure 4**). The greater number of endemic species may indicate that the survival conditions required by these liverworts are harsh and that only the local area can provide the most suitable survival conditions, which reflects that these areas are among the diversity centers of liverworts. As shown in **Figure 5**, the geographical distribution patterns of national endemic and provincial endemic liverwort showed high species richness in the central areas of liverwort species diversity, but there were differences in individual provinces. The species richness of endemic liverwort in China is highest in southwestern inland provinces, whereas that of provincial endemic liverwort in China is highest in southwestern Yunnan Province and coastal island areas in Taiwan and Hainan Provinces.

## 4 Discussion

### 4.1 Availability of data

Due to the data come from different sources, the quality of each liverwort inventory varies with the age, scope, and concept of classification of the documentation. Older lists indicate outdated taxon names and occasionally list several synonymous names at once. In addition, lists are compiled in different ways, and different authors adhere to different taxonomic concepts; thus, it is important to standardize the data via reliable reference species lists. The ColChina database has proven to be an important species resource in China for this purpose. Under the auspices of the Commission for Biological Diversity of the Chinese Academy of Sciences, the COLChina Database collates and verifies the taxonomic information of all biological species distributed in China, establishes and maintains the COLChina Database, provides free services for users all over the world, and updates an annual edition. The list contains data on the scientific name, synonym, alias, source of literature, classification system and distribution area of each species, as well as the contents of the Chinese name and the Hanyu pinyin of the Chinese name. The 2024 edition of the List of Biological Species of China was updated in May 2024, and a total of 3,064 reliable liverwort data points were obtained from the database, including 1,192 scientific names and 1,872 synonymous names. Notably, this study removed four liverworts from the ColChina database, including *Porella longifolia* and *Heteroscyphus acutangulus*, which have been proven to be misidentified; *Treubia insignis*, which lacks reliable evidence of its distribution in China*;* and *Tritomaria quinquedentata*, which has problems related to the duplication of species names.

Specimens provide important evidence for checking information on the distribution of liverwort species in the process of species cataloging, but the quality of the information varies. For liverwort without specimen evidence, records or other information are considered to be more reliable and should be retained, which may lead to an overestimation of species. Moreover, when addressing “heteronyms” in the list, we should focus on the species information contained in the Latin names. According to the naming principles of the international Code of Nomenclature, for species that have problems with changes in Latin names, we should review the original literature and verify the Tropicos and other databases to determine whether they are the same species. In addition, the Tropicos database was used to carry out name correction, in which the retrieved information included the naming status of each given species. In this study, most species names in the liverwort list were shown to be accepted, and some species names were disputed, however, the species derived from a real and reliable source were retained.

Judging from the completeness and accuracy of the liverwort data, the current understanding of liverwort species is still insufficient. The richness of liverwort species in some areas depends on local surveys, and the presence of species diversity in inadequately surveyed areas is expected to be significantly underestimated. In addition, data from inventories and flora are not without problems (Kreft et al., 2010). At the same time, taxonomic revisions of possible taxa are still lacking (Mutke and Geffert, 2010). However, the offsetting effects of increased sampling and increased synonyms tend to result in relatively constant species numbers in these areas, which makes liver-geographic area studies feasible; some of the documented patterns of species richness are predictable and likely to reflect real-world biogeographic patterns, and uneven survey areas are evident, which provides important theoretical data for background surveys and assessments of priority areas and national strategic areas for the conservation of liverwort biodiversity. As a result, the current dataset is relatively reliable and informative for research purposes.

### 4.2 Life science identifiers

Owing to the wide range of latitudes and latitudes in China and the large variations in climate (e.g., from tropical rainforests in southernmost China to boreal forests in northernmost China and deserts in western China) (Su et al., 2020), they have been shown to be highly adaptable to different environmental conditions. However, at the same time, species richness per unit area varies widely across provinces in China. Although the vast majority of China has a temperate climate, almost all of the regions with high species richness are in tropical or subtropical climates. China’s humid tropical and subtropical mountains have not only been a hotspot for liverwort diversity but also a cradle of a high degree of bryophyte diversity in recent years and a refuge for many monotypic and oligotypic genera of bryophytes (Yin et al., 2021). Some botanist reported that there is a significant latitudinal gradient in the species richness of global moss diversity. In tropical regions, Lejeuneaceae strongly affects the species diversity distribution of liverwort, and the key factor may be adaptation to the leaf-attached habitats dominated by this group (von Konrat et al., 2008). In addition, studies have shown that the percentage area, precipitation range and temperature range of nature reserves in each region are strongly correlated with regional species richness changes (Song et al., 2021). When individual environmental variables are considered, on average, precipitation-related variables are more strongly correlated with moss species richness and species density than are temperature-related variables (Qian and Chen, 2016). Water availability is the most important climate predictor for bryophytes and an important climate factor affecting the distribution patterns of liverwort species. Many dryland areas also have low species richness, which indicates that they are highly dependent on freely available water, such as precipitation or air water (Su et al., 2020). Only a few specialized taxa have been able to reduce this dependence on water and thus be able to survive in dryland areas (Frahm, 2001). The above mention hot spot centers of liverworts and some factors affecting the distribution pattern of liverworts, most liverworts have the characteristics of warm and humid, resulting in tropical or subtropical areas, and where precipitation is sufficient, the most suitable place to survive. Only a few liverworts can adapt to cold and arid areas. Therefore, due to the gradual formation of the distribution center of liverworts due to local climatic factors, it can also explain the huge differences in the distribution patterns of different groups. The overall distribution pattern of liverworts is highly similar to that of official databases and published studies in recent years. However, the current data provided in this study provide a more detailed description of the distribution pattern of liverwort species richness in China than any previous study, and provide relatively non-conservative data for various provinces. The present data provide a more detailed description of the distribution patterns of liverwort species richness in China than does any previous comparable study.

### 4.3 Endemic phenomena of Chinese liverworts

China’s vast territory, diverse climate types and rich habitat types provide favorable conditions for the distribution of endemic liverwort. The endemic phenomenon is the result of the interaction between the genetic characteristics of the original population in each region and their specific natural conditions and can be studied at various taxonomic levels, such as the family, genus and species levels (Zhang, 1997; Chen et al., 2011). There are 155 liverwort species in 28 families and 42 genera endemic to China, with more than 10 species in each family. Only 4 families were the dominant endemic families. For example, Frullaniaceae, Lejeuneaceae, Plagiochilaceae and Solenostomataceae accounted for 56.13% of the total endemic species. There are more than 10 species within the genus, and 4 genera are endemic dominant genera of Chinese liverwort, such as *Frullania*, *Plagiochila*, *Cololejeunea* and *Solenostoma*, accounting for 48.39% of the total endemic species. The above endemic families and genera are also the dominant families and genera of the whole liverwort group in China, which may be due to the large species-level differentiation of the dominant groups. Furthermore, for the dominant families and genera, the adaptability to the environment may be more stable than other groups. Some species in the dominant families and genera have gradually become endemic species because they can only adapt to the environment of a certain area. There are two assumptions: One is that it only survives in this region and has no opportunity to grow in areas close to the same latitude environment. Second, there are opportunities and have tried similar areas of the environment, but failed to survive. Species endemism is a concept of geographical regions, and if it occurs only in a certain region, it can be considered endemic to that region (Anderson, 1994). In general, the distribution center of endemic liverworts in China is mainly concentrated in the important mountains and rivers in China. The special topography creates unique climatic conditions, which gradually evolves into the distribution center of species and breeds extremely rich liverworts. Climate fluctuations can cause plant migration along latitudes or altitudes and lead to changes in the distribution range of plants on continental margins and islands. This may lead to repeated isolation and contact between different plant populations, which may promote or limit population differentiation and speciation, thus affecting the formation and distribution of regional species diversity and speciation (Jiang, 2022). Therefore, it can explain the reason why there are many endemic species in coastal areas and islands. There are many endemic species in Yunnan-Guizhou Plateau, Sichuan Basin, Himalayas, Qinling Mountains, Wuyi Mountains, Taiwan Mountains and Changbai Mountains. The number of endemic species is also the largest in Yunnan Province, Taiwan Province and Hainan Province, and its species distribution is more narrow. Due to the narrow distribution area of endemic plants, they are prone to extinction (Lamoreux et al., 2006). Therefore, studies on plant endemism can provide a certain theoretical basis for the priority protection of regional species diversity.

## 5 Conclusion

Detailed data on the distributions of individual species are limited, especially in tropical or subtropical regions, and identifying priority areas of liverwort diversity seems to be the most feasible approach. The results revealed that the centers of liverwort diversity and endemic species differentiation were concentrated in the southern Himalayas, Hengduan Mountains, Yunnan-Guizhou Plateau and Sichuan Basin in the southwest, the Qinling Mountains to the Taihang Mountains and Changbai Mountains in the north, the Xiaoxing’an Mountains, and the coastal areas of the middle and lower reaches of the Yangtze River to the Taiwan Mountains in the south. There are great differences in the distribution patterns of major liverwort groups. The distribution patterns of the large liverwort groups are roughly the same as the overall distribution patterns of liverwort in China, whereas the distribution patterns of the small- and medium-sized liverwort groups are more diverse. The distribution centers of liverwort do not necessarily coincide with the overall biodiversity centers, and these places need to pay special attention to the status of rare species and threats through local assessment and carry out targeted conservation actions in unprotected areas.

## Data Availability

The original contributions presented in the study are included in the article/**Supplementary Material**. Further inquiries can be directed to the corresponding authors.
