# Supplementary material for: Liverwort diversity in China: spatial and taxonomic patterns on species richness
Source: Front Plant Sci. 2024 Dec 19;15:1513221. doi: 10.3389/fpls.2024.1513221 (PMC11693448; doi:10.3389/fpls.2024.1513221)
Supplement: Supplementary file 1 [file DataSheet1.docx]

Supplementary Material

# Supplementary Data

Literature sources used in documenting province-level distributions of liverworts in this study.

[1] Ren, N., Xu, J. (2024) Research Progress of Bryophyte Flora in Inner Mongolia from 2012 to 2022. Journal of Inner Mongolia Normal University（Natural Science Edition）.53(02):129-139. DOI:10. 3969/j. issn. 1001-8735. 2024. 02. 003.

[2] Ma, X. Y., Xiang, Y. T., Zheng, X., Wu, Y. H. (2024). *Saccogynidium* (Acrobolbaceae, Hepaticae), a genus new to ZhejiangjJournal of Hangzhou. Normal University (Natural Science Edition).23(03):270-272+322. DOI: 10.19926/j.cnki.issn.1674-232X.2023.05.021.

[3] Song, X.; Gu, J.; Ye, Y.; Li, W.; Liao, Y.; Wang, R.; Ma, H.; Shao, X. The Diversity and Community Pattern of Liverworts on Sygera Mountain, Tibet. Forests 2024, 15, 48. DOI: 10.3390/f15010048

[4] Chen, X., Tu, S.W., Dai, Z., Gao, S., Wang, Y. F., Xing, S. C., Wei, B. J., Tang, L. Y., Shi, R. P., Wang, X. R., Liu, Y. Y., Zhao, D. P., Tang, X., Yao, X., Zhao, M. S., Wu, H. X., Qi, X. B., Zhang, J., Li, M., Wang, J. (2023). Bryophytes diversity of Tianmushan National Nature Reserve, Zhejiang Province. Biodiversity Science, 31, 22649. DOI: 10.17520/biods.2022649.

[5] Liang, L. W., Nurdin·Turgun，Sulayman·Mamtimin.，Wang, P. J., Li, W. (2023). Additions to the liverworts in Xinjiang. Plant Science Journal. 41（5）：563−572. DOI:10.11913/PSJ. 2095-0837. 23013.

[6] Huang, P., Tang, Q. M., Xiang, Y. L., He, W. C., Wang, S. L., Wei, Y. M. (2023). Ten species of liverworts and hornworts new to Guangxi, China. Guihaia. 1-14. DOI:10.11931/guihaia.gxzw202304060.

[7] Wang, C. (2023). The development characteristics and formation mechanism of saxicolous bryophyte crust in the northern foothills of Qinling Mountains. Northwest Agriculture and Forestry Technology University. Dissertation of Doctor.

[8] Hong, J. K., Song, X. T., Gu, J. Q., et al. (2023). Calyculariaceae，a newly recorded family of bryophytes in Tibet，China. Journal of Plant Ｒesources and Environment.32(05):95-97. DOI: 10．3969/j．issn．1674－7895．2023．05．11.

[9] Song, Z. Q., Ye, W., Dong, S. Y., et al. (2023). A dataset on inventory and geographical distributions of higher plants in Guangdong, China. Biodiversity Science. 31, 23177. DOI: 10.17520/biods.2023177.

[10] Ling, C. (2023). History of bryophytes collection and research in South China before 1949. Inner Mongolia Normal University. Dissertation of Master.

[11] Wu, L., Chen, S. L., Ai, X. R., et al. (2023). Bryophytes in Mulinzi National Nature Reserve, Hubei Province, China. Beijing: Science Press.

[12] Deng, X. D. (2023). Study on Bryophyte Flora and Distribution Pattern in Yudaokou area of Hunshandake Sandy Land. Inner Mongolia Normal University. Dissertation of Master.

[13] Li, Y. S. (2023). Bryophyte diversity and its influencing factors in temperate deciduous broad-leaved forest in Funiu Mountain. Henan Agricultural University. Dissertation of Master.

[14] Tan, H. Y., Xia, F. M., Cao, W. (2023). Preliminary Study on Bryophytes in Aha Lake National Wetland Park in Guiyang City, Guizhou Province. Journal of Mountain Agriculture and Biology. 42(05):88-92. DOI: 10.15958/i.cnki.sdnyswxb.2023.05.016.

[15] Xu, G. L., Li, X. J., Zhang, C. Y. (2023). New Data of Bryophyte Distribution in Jiulianshan National Nature Reserve, Jiangxi Province. Biological Disaster Science. 46(01):60-65. DOI: 10.3969/j.issn.2095-3704.2023.01.11.

[16] Zhang, H., Tao, J. W., Cheng, H., et al. (2023). Bryophytes Flora in the Northern Slope of Mt. Dabie, Anhui Province. Acta Bot.Boreal. - Occident.Sin. 43(02):335-342. DOI: 10.7606/j.issn.1000-4025.2023.02.0335.

[17] Tao, J. W., Tang, G. T., Cheng, Q., Shi, X. Q. (2023). Nine newly recorded species of liverworts in Anhui Province. Journal ofPlant Resources and Environment. 32(01):98-100. DOI: 10.3969/i.issn.1674-7895.2023.01.13.

[18] Xiang, Y. L., Zhu, R.L. (2023). "Morphological and Molecular Evidence for a New Species, *Mannia gradsteinii* sp. nov. (Aytoniaceae) from Southwestern China," Cryptogamie, Bryologie. 44(12), 237-245. DOI:10.5252/cryptogamiebryologie2023v44a12.

[19] Zheng, T. X., Long, D. G. (2023). A contribution to the knowledge of selected Marchantiaceae taxa in China. Journal of Bryology, 45(2), 96–104. DOI:10.1080/03736687.2023.2232668.

[20] Promma, C., Lu, S. H., Shen, C., Zhang, L. N., Zhu, R. L. (2023). Range extension, taxonomic note, molecular-phylogenetic relationship, and conservation of *Frullania iriomotensis* S.Hatt. (*Marchantiophyta*, Frullaniaceae), a rare liverwort previously known only from the type locality in Japan. Phytotaxa. 589: 1-21. DOI: 10.11646/PHYTOTAXA.589.1.5.

[21] Ma, X. Y., Zhang, F. Y., Huang, W. Z., et al. (2022). Fossombroniaceae, a New Record Family of Liverworts from Zhejiang Province. Journal of Hangzhou Normal University (Natural Science Edition). 21(06):590-594. DOI: 10.19926/i.cnki.issn.1674-232X.2022.06.006.

[22] Chen, C. F., Zhang, Z. H., Tu, K., Sun, Y. (2022). New Records of Bryophytes in Jiangxi. Chinese Wild Plant Resources. 41(10):81-83+106. DOI: 10.3969/j.issn.1006-9690.2022.10.015.

[23] Jin, M. Z., Ren, Z. J., Zhao, C. G., Lin, J., Hou, J. H., Xu, Y. K. (2022). New Records for Zhejiang Bryophyte Flora. J. Anhui Agric. Sei. 50(13):119-122+132. DOI: 10.3969/i.issn.0517-6611.2022.13.031.

[24] Sheng, W., Huang, W. Z., Zhang, F. Y., Yu, Z. C., Wu, Y. H. (2022). New Records of Bryophytes in Zhejiang Province, China. Journal of Hanazhou Normal University (Natural Science Edition). 21(01):31-39+87. DOI: 10.19926/i.cnki.issn.1674-232X.2022.01.005.

[25] Shi, R. P., Xing, S. C., Tang, X., Tu, S. W., Wang, Y.F., Wang, J. (2022). New records of liverwort species in Zhejiang Province. Journal of East China Normal University (Natural Science). (01):62-69. DOI: 10.3969/j.issn.1000-5641.2022.01.008.

[26] Liao, Y. J., Song, X. T., Li, W., et al. (2022). Two newly recorded thalloid liverworts in Tibet, China. Journal of Central China Normal University (Natural Science Edition). 56(03):481-486. DOI: 10.19603/j.cnki.1000-1190.2022.03.015.

[27] Zuo, J. R. (2022). Diversity of bryophytes in the northern mountains of Yanshan. Inner Mongolia Normal University. Dissertation of Master.

[28] Xu, G. L., Yuan, X. H., Li, Z. L., Zeng, X. H., Hu, X. H., Liu, J. (2022). New Records of Bryophytes in Jiulianshan Nature Reserve of Jiangxi Province. Subtropical Plant Science. 51(06):489-492. Doi: 10.3969/j.issn.1009-7791.2022.06.009.

[29] Zhang, S. M., Li, W., Li, D. N. (2022). Inventory of species diversity of Liaoning higher plants. Biodiversity Science. 30, 22038. DOI: 10.17520/biods.2022038.

[30] Gu, R. (2022). Bryophyte diversity in the Qilian Mountain region in Qinghai. Inner Mongolia Normal University. Dissertation of Master.

[31] Wang, R. H., Yu, Z. C., Jin, W. (2022). Biodiversity of Xianxialing Nature Reserve. Hangzhou: Zhejiang University Press.

[32] Ren, Z. J., Xu, Y. K., Zhao, C. G., et al. (2022). Bryophytes in Jingning. Hangzhou: Zhejiang University Press.

[33] Liu, F., et al. (2022). Comprehensive scientific investigation report of Hubei Wufenghouhe National Nature Reserve. Beijing: China Forestry Press.

[34] Tu, S. W. (2022). Elevational patterns of bryophyte diversity in West Tianmu Mountain of Zhejiang Province. East China Normal University. Dissertation of Master.

[35] Tian, Y. X. (2022). A Study on the Diversity Within the Bryoflora of Motuo, Xizang. ShanDong Normal University. Dissertation of Master.

[36] Bi, X. F. (2022). Molecular phylogeny of *Riccardia* (Aneuraceae). East China Normal University. Dissertation of Master.

[37] Zhang, H., Shi, X. Q., Cheng, X. F. (2022). Species diversity of Plagiochila in Anhui Province. Journal of East China Normal University (Natural Science). (06):130-138. DOI: 10.3969/j.issn.1000-5641.2022.06.013.

[38] Cheng, H., Tao, J. W., Zhang, H., Shi, X. Q. (2022). Characteristics of bryophyte flora of Yuexi County in Anhui Province. Journal of Plant Resources and Environment. 31(05):81-91. DOI: 10．3969/j．issn．1674－7895．2022．05．10.

[39] Chen, C. F., Zhang, Z. H., Tu, K., et al. (2022) Study on the Flora of Bryophyte in Jiulingshan National Nature Reserve, Jiangxi Province, China. Acta Bot. Boreal. - 0ccident. Sin.42(07):1239-1247. DOI: 10.7606/i.issn.1000-4025.2022.07.1239.

[40] Xing, S. C., Tang, L. Y., Dai, Z., et al. (2022). Bryophyte diversity in Shitai County and Qingyang County, Anhui Province. Biodiversity Science. 30, 21186. DOI: 10.17520/biods.2021186.

[41] Dai, Z., Chen, X., Zhang, J. H., et al. (2022) Species diversity of epiphyllous liverworts and host plants in the Wuyanling National Nature Reserve, Zhejiang Province. Biodiversity Science. 30, 21229. DOI: 10.17520/biods.2021229.

[42] Zhou, X. P., Tang, L. Y., Xia, H. X., et al. (2022). Bryoflora Characteristics in Niangniang Mountain National Wetland Park, Liupanshui, Guizhou. Journal of Tropical and Subtropical Botany. 30(01):111-124. DOI: 10.11926/jtsb.4414.

[43] Akida, A., Li, W., Mamtimin, S. (2022). Taxonomy and distribution of *Frullania Raddi*. in Xinjiang, China. Journal of Central China Normal University (Natural Science Edition). 56(02):290-296. DOI: 10.19603/j.cnki.1000-1190.2022.02.012.

[44] Shi, X. Q., Wang, J. (2021). Bryophyte checklist of Anhui Province, China. Biodiversity Science, 29, 798-804. DOI: 10.17520/biods.2020314.

[45] Jia, Y., He, Q., Wu, P. C., et al. (2021) Flora of the Liverworts and Hornworts of Qinling Mountains, China. Beijing: Science Press.

[46] Guo, L. (2021). Niche Characteristics of Bryophyte Community in Xiaoginling and Baotianman. Henan Agricultural University. Dissertation of Master.

[47] Li, F. (2021). Study on Flora of Bryophytes in Guizhou Chishui Alsophila National Nature Reserve. Guizhou Normal University. Dissertation of Master.

[48] Huang, W. Z. (2021). Studies on diversity and ecology of bryophytes in the periglacial zones. Hangzhou Normal University. Dissertation of Master.

[49] Zhang, L. N., et al. “*Radula subacuminata*, a new epiphyllous species of Radula (Marchantiophyta) from China and Vietnam.” The Bryologist. 124 (2021): 257 - 270. DOI: 10.1639/0007-2745-124.2.257.

[50] Li, L. (2021). Study on Classification and Environmental Adaptability of bryophyte in Mu Us Sandy Land. Inner Mongolia Normal University. Dissertation of Master.

[51] Qin, X. T. (2021). Flora analysis of bryophytes in Limushan Nature Reserve, Hainan, China. Hainan University. Dissertation of Master.

[52] Bai, Y. (2021). Species diversity of bryophytes in Qianjiangyuan National Park. East China Normal University. Dissertation of Master.

[53] Zhang, M. Q. (2021). Taxonomical revision and phylogenetic study of *Calypogeia* Raddi in China. East China Normal University. Dissertation of Master.

[54] Chen, Y. Q. (2021). A taxonomical revision of *Pleurozia*. East China Normal University. Dissertation of Master.

[55] Ren, Q. Q. (2021). Interaction Mechanism Between Epilithic Mosses Functional Traits and Habitat in Typical Karst Area. Guizhou University. Dissertation of Master.

[56] Li, Y.S.，Xiao, M.，Zhang, H.P.，Shao, Y.Z.，Liu, F.Q.，Yuan, Z.L.，Chen, Y． (2021). Effects of different growth substrates on phylogenetic diversity of bryophytes in Baiyunshan National Forest Park． Plant Science Journal,39 (6): 600-609. DOI: 10. 11913/PSJ. 2095-0837. 2021. 60600.

[57] Quan, D. L., Yang, B., Ma, W. Z., et al. (2021). Bryophyte diversity and its threat status in Xishuangbanna. Biodiversity Science, 29, 545-553. DOI: 10.17520/biods.2020216.

[58] Zhang, L. N., Promma, C., Shu, L., Wei, Y. M., Wang, J., Do, T. V., Lu, T. N., Zhu, R. L. (2021) "*Radula subacuminata*, a new epiphyllous species of Radula (Marchantiophyta) from China and Vietnam," The Bryologist .124(2), 257-270. DOI: 10.1639/0007-2745-124.2.257

[59] Dai, Z., Xing, S. C., Gradstein, S. R., Chen, X., Zhu, R. L., Wang, J. (2021). "New species or infraspecific variation? A case study of *Ptychanthus striatus* var. *motuoensis* var. nov. (Marchantiophyta: Lejeuneaceae) from Xizang, China," The Bryologist. 124(4), 475-483. DOI: 10.1639/0007-2745-124.4.475.

[60] Potemkin, A. D., Vilnet, A. A., Bakalin, V. A. (2021). A case of morphological convergence in sections Stephania and Ciliatae of the liverwort genus *Scapania* (Scapaniaceae, Marchantiophyta) and an extension of the range of *S. metahimalayana* to China. Journal of Bryology. 43(4), 339–346. DOI: 10.1080/03736687.2021.2010001.

[61] Tang, Q. M., Wei, Y. M., Liu, Y., Wei, X., Tang, J. M. (2020). Eight New Recorded Species of Bryophytes in Guangxi. Journal of Guangxi Academy of Sciences. 36(01):30-36. DOI: 10.13657/j.cnki.gxkxyxb.20200317.003.

[62] Cheng, Q., Cheng, H., Zhang, H., Shi, X. Q. (2020) Two new record species of hepaticae distributed in Anhui Province. Journal of Plant Resources and Environment. 29(01):78-80. DOI: 10．3969/j．issn．1674－7895．2020．01．12.

[63] Cao, W. (2020). A taxonomic study of Marchntiophyta in the East Yunnan-Guizhou Plateau. Guizhou University. Dissertation of Doctor.

[64] Cheng, Q. (2020). The Bryoflora of Southern Anhui Province. Anhui Normal University. Dissertation of Master.

[65] Hong, L. (2020). Study on Diversity and Floristic Geography of Bryophyte in Qingjiang River Basin. Hubei University for Nationalities. Dissertation of Master.

[66] Zhang, W. P. (2020). Systematics studies of *Scapania* (Dumort.,) Dumort. in China. East China Normal University.

[67] Li, Y.Y. (2020). Diversity and Biogeography of bryophytes in Haitan Island and its adjacent Islands, Fujian Province. Shanghai Normal University. Dissertation of Master.

[68] Fan, P. Z. (2020). Effects of Environmental Factors on the Diversity and Distribution of Bryophytes in Baotianman National Nature Reserve. Henan Agricultural University. Dissertation of Master.

[69] Yi, X.B. (2020). A taxonomical revision of the Aneuraceae of China. East China Normal University. Dissertation of Master.

[70] Shen, L. (2020). Studies on flora and geographic distribution of bryophytes in Dongchong peninsula and it’s adjacent islands, Fujian Province, China. Shanghai Normal University. Dissertation of Master.

[71] Tian, F. (2020). Studies on Flora of Higher Plants in Wetlands of Guilin. Guangxi Normal University. Dissertation of Master.

[72] Promma, C. (2020). Taxonomy and Phylogeny of the genus *Radula* subgenus *Amentuloradula* Devos et al. (Radulaceae, Marchantiophyta). East China Normal University. Dissertation of Master.

[73] Mao, R. K., Hu, J.H. (2020). Preliminary Study on Bryophyte Community in Xiaolongshan Forest Region. Qinghai Agriculture and Forestry Science and Technology. (04):32-35+84.

[74] Luo, Y. X., Tang, Q. M., Xue, Y. X. (2020). Study on Diversity Charateristics of Bryophytes in Chuandong Tiankeng，Guangxi. Journal of Guangxi Normal University (Natural Science Edition). 38(05):104-111. DOI: 10.16088/j.issn.1001-6600.2020.05.013.

[75] Bai, Y., Chen, S. W., Qian, H. Y., et al. (2020) Species diversity of epiphyllous liverworts in Qianjiangyuan National Park, Zhejiang. Biodiversity Science, 28, 231-237. DOI: 10.17520/biods.2019273.

[76] Cheng, Q., Shi, X. Q. (2019). Haplomitriaceae，a new recorded family in Anhui Province. Journal of Biology. 36(02):59-60. DOI: 10. 3969/j. issn. 2095-1736． 2019. 02. 059.

[77] Song, X. T., Fan, Y. J., Li, W., Ma, H. P., Shao, X. M. (2019) New records of three species in Calypogeiaceae distributed in Tibet. Journal of Plant Resources and Environment. 28(03):117-119. DOI: 10． 3969/j． issn． 1674-7895． 2019． 03． 15.

[78] Tang, X. (2019). Diversity Research of Liverworts and Hornworts in Huangshan-Tianmu Mountains Range and Xianxia-Wuyi Mountains Range in East China. East China Normal University. Dissertation of Master.

[79] Huang, L. L. (2019). Studies on the liverwort flora of Sino-Vietnam Border Area. East China Normal University. Dissertation of Master.

[80] Tengjing, J. Z. (2019). Bryophyte Illustrated Handbook. Beijing: China Light Industry Press.

[81] Wei, W. (2019). Studies on flora and geographic distribution of bryophytes in Daishan island and the nearby islands, Zhoushan, Zhejiang. Shanghai Normal University. Dissertation of Master.

[82] Shaanxi Foping National Nature Reserve Management Bureau. (2019). Collection of Research Papers on Biodiversity of Foping National Nature Reserve in Shaanxi. Yangling: Northwest A & F University Press.

[83] Gulnigar, A., Turgun, N., Dilnur, A., Mamtimin, S. (2019). Research on bryophyte community type in Tomur Peak National Natural Reserve. Journal of Central China Normal University (Natural Science Edition). 53(04):534-541. DOI: 10.19603/j.cnki.1000-1190.2019.04.013.

[84] Yang, W. C., Ran, J. C., et al. (2019). Guizhou Jin 'an Longyin broad-leaved forest state nature reserve comprehensive scientific expedition set. Beijing: China Forestry Press.

[85] Zhang, L. N. (2019). Systematies studies of *Radula* Dumort. in China. East China Normal University. Dissertation of Master.

[86] Shu, L. (2019). Phylogeny and taxonomy of Leptolejeunea (Lejeuneaceae, Marchantiophyta). East China Normal University. Dissertation of Master.

[87] She, X. J. (2019). Study on the Bryoflora and Diversty of bryophytes in Qizimei mountain National Nature Reserve，Hubei province. Hubei Minzu University. Dissertation of Master.

[88] Li, Y. J. (2019). A taxonomic revision of Chinese Cololejeunea. East China Normal University. Dissertation of Master.

[89] Huang, H. (2019). Characteristics of bryophyte communities and their accumulation of metals in Karst Bauxite Mining Area. Guizhou Normal University. Dissertation of Master.

[90] Liu, Y., Yang, Y. S. (2019) Importance of conservation priority areas for bryophyte biodiversity in Chongqing. Biodiversity Science. 27, 677-682. DOI: 10.17520/biods.2019045.

[91] Cao, W., Xiong, Y. X., Zhao, D.G., Qu, J. J., Tan, H. Y. (2019). “*Porella perrottetiana* var. *angustifol*ia （Porellaceae， Marchantiophyta）, new to China, and taxonomic revision of *Porella perrottetiana* in the East Yunnan-Guizou Plateau” Herzogia. 32(2), 344-356. DOI: 10.13158/heia.32.2.2019.344.

[92] Tian, Y. X., Ren, S. J., Wang, C. X., Zhao, Z. T. (2018) *Heteroscyphus Schiffn*． —a new record of gerus bryophyte to Shandong Province. Shandong Science.31(03):108-109. DOI: 10．3976/j．issn．1002-4026．2018．03．016.

[93] She, X. J., Liu, X. F., Hong, L., et al. (2018). A New Checklist of Liverworts in Hubei Province. Hubei Agricultural Sciences. 57(23):109-117. DOI: 10.14088/j.cnki.issn0439-8114.2018.23.026.

[94] Song, X. T., Shao, X. M., Sun, Y., Jiang, Y. B., Song, S. S., Liu, X. C., Wang, Q. G. (2018) Research on the bryoflora of Dongling Mountain, Beijing, China. Plant Science Journal. 36(4): 554-561. DOI: 10. 11913/PSJ. 2095－0837. 2018. 40554.

[95] Zang, C. (2018). Studies on flora and geographic distribution of bryophytes in Zhoushan island and the nearby islands, Zhejiang Province. Shanghai Normal University. Dissertation of Master.

[96] Tan, H. Y. (2018). Study on Bryophytes Species Diversity in Karst Ravine from Guizhou Province. Guizhou University. Dissertation of Master.

[97] Zhu, R., Shu, L., He, Q., Wei, Y. (2018). *Soella* (Marchantiophyta: Lejeuneaceae), a new genus from China and Japan. The Bryologist. 121, 324-339. DOI: 10.1639/0007-2745-121.3.324.

[98] Cheng, L. Y. (2018). Study on flora and geographic distribution of bryophytes in Qingliangfeng National Nature Reserve, Zhejiang Province, China. Shanghai Normal University. Dissertation of Master.

[99] Yan, L. C. (2018). Studies on flora and geographic distribution of bryophytes in Ma’an archipelago, Zhoushan, Zhejiang. Shanghai Normal University. Dissertation of Master.

[100] Cai, J. R. (2018). Studies on flora and geographic distribution of bryophytes in Shengsi archipelago, Zhoushan, Zhejiang. Shanghai Normal University. Dissertation of Master.

[101] Weng, T., Huang, W. P., He, Q. Q., et al. (2018). Bryophyte Species Diversity of Alsophila Community in Chishui Alsophila National Nature Reserve. Subtropical Plant Science. 47(04):339-344. Doi: 10.3969/j.issn.1009-7791.2018.04.007.

[102] Tang, Q. M., Cheng, X. F., Wei, Y. M. (2018). Study on Species Diversity of Epiphyllous Liverworts in Karst Region of Guangxi-Vietnam Border. Journal of Tropical and Subtropical Botany. 26(05):481-489. DOI: 10.11926/jtsb.3870.

[103] Qian, K., Bi, X. F., Shu, L., Zhu, R. L. (2018). *Porella longifolia* (Steph.) S.Hatt. and *Porella densifolia* var. *robusta* (Steph.) S.Hatt. (Porellaceae, Marchantiophyta) excluded from the liverwort flora of China. Phytotaxa. 350 (2): 182–186. DOI: 10.11646/phytotaxa.350.2.9.

[104] Wang, Y. (2017). Studies on Taxonomy and Flora of Hepaticae and Anthocerotae in wudalianchi Volcanosheilongjang, China. Inner Mongolia University. Dissertation of Master.

[105] Hou, R. (2017). Epiphytic Bryophytes Species Diversity of Forest Ecological Systems in the Ancient Rock Stream Periglacial Landforms of Liaoning Eastern Mountains. Liaoning Normal University. Dissertation of Master.

[106] Wu, P. C., et al. (2017). Chinese Illustrated Bryophytes. Beijing: China Forestry Press.

[107] Chen, W., Qu, Z. C., Zhang, Y., et al. (2017). Biodiversity of Baishilazi National Nature Reserve. Shenyang: Liaoning Science and Technology Press.

[108] Wang, W. H., et al. (2017). Field Practice Course of Higher Plants in Beijing. Beijing: China Forestry Press.

[109] Cui, Z. N. (2017). Study on Taxonomy of Jungermanniales Complex in Guizhou. Guizhou University. Dissertation of Master.

[110] Liu, S. L. (2017). Study on Biodiversity of Bryophytes in Kuankuoshui Nature Reserve. Guizhou University. Dissertation of Master.

[111] Li, Y. (2017). Study on the Bryoflora and Diversity of Gaogesitai Hanwula National Nature Reserve. Inner Mongolia Normal University. Dissertation of Master.

[112] Li, Y., Dong, Y. Z., Li, W. Z., et al. (2017). Diversity Research of the Bryophyta on the Wall of Tongwan Castle Site. Chinese Wild Plant Resources. 36(02):61-65.

[113] Zhang, L., Jia, Y., Mao, L. H. (2016). Field Guide to Wild Plants of China. Beijing: Commercial Press.

[114] Editorial Committee of Higher Plants of China in Colour. (2016). Higher Plants of China in Colour Volume I Bryophytes. Beijing: Science Press.

[115] Cao, W. (2017). Study on Spatial Distribution of Bryophytes in Miaoling Mountains of Guizhou, China. Guizhou University. Dissertation of Master.

[116] Guzalnur, A. (2016). Flora and Taxnomy of Liverworts in Eastern Tianshan,Xinjiang. Xinjiang University. Dissertation of Master.

[117] Gun, F. R. (2016). Studies on Biodiversity of Bryophytes in Wuyi Mountain, China. Hangzhou Normal University. Dissertation of Master.

[118] Wang, T. Y. (2016). Studies on diversity and ecology of bryophytes in the headwaters region of Urumqi River. Hangzhou Normal University. Dissertation of Master.

[119] Wu, W. X. (2016). The study on species and floristie characteristies of wetland plant in Guangxi, China. Guangxi Normal University. Dissertation of Master.

[120] Zhang, X. Y. (2016). Chinese reindeer area bryophyte diversity and Ecological impact of reindeer habitat communities. Inner Mongolia Normal University. Dissertation of Master.

[121] Thorbiyamu, U., Serik, T., Bumai, T. S., (2016). Newly recorded species of Fossombroniaceae and Hygrobiellaceae from Xinjiang——*Fossombronia pusilla* (L.) Dumort. And *Hygrobiella laxifolia* (Hook.) Spruce. Journal of HuaZhong Normal University (Natural Sciences). 50(04):588-591. DOI: 10.3969/j.issn.1000-1190.2016.04.019.

[122] Tang, Q. M., Xue, Y. G., Dang, G. L., et al. (2016). Bryophytes Species New to Guangxi, China. Journal of Guangxi Normal University (Natural Science Edition). 34(02):143-146. DOI: 10.16088/j.issn.1001-6600.2016.02.021.

[123] Wang, Y., Zhao, Y., Song, L., et al. (2016). Morphological Characteristics and Geographical Distribution. Acta Bot. Boreal. - Occident. sin. 36(03):618-623. DOI: 10.7606/i.issn.1000-4025.2016.03.0618.

[124] Liu, Y., Pi, C. Y., Tian, S. (2016) Bryophyte biodiversity of the Dabashan National Nature Reserve in Chongqing. Biodiversity Science. 24, 244-247. DOI: 10.17520/biods.2015236.

[125] Zhang, L. N., Zhu, R. L. "*Radula hainanensis* (Radulaceae), a new species from China," The Bryologist, 119(1), 52-59. DOI: 10.1639/0007-2745-119.1.052.

[126] Tian, H. L., Wang, W. H., Li, J. Q., et al. (2015). Bryophyte Diversity: Beijing Baihuashan Nature Reserve. Beijing: China Forestry Education Press.

[127] Zhou, S. Q. (2015). Study on the species diversity of bryophytes on the southern slope of Miaoling, Guizhou. Guizhou University. Dissertation of Master.

[128] Tang, F. L., Du, F., Sun, G. Z., et al. (2015). Yunnan Nangunhe National Nature Reserve Comprehensive Scientific Research. Beijing: China Forestry Press.

[129] Cong, M. C. (2015). A preliminary study on liverworts of Mts. Taihang and Yanshan in Hebei. Hebei Normal University. Dissertation of Master.

[130] Sa, R. L. (2015). A Study on the Diversity within the Bryoflora of Southern Greater Khingan Mountains, China. Inner Mongolia University. Dissertation of Master.

[131] Zhi, J. Z. (2015). Preliminary Study on bryophytes in Longzhou County of Guangxi. Central China Normal University. Dissertation of Master.

[132] Tang, Y. X. (2015). Studies on flora and geographic distribution of bryophytes in Shiwandashan natural reserve, Guangxi Province, China. Shanghai Normal University. Dissertation of Master.

[133] Jia, H. L. (2015). The Effect of Spatial Heterogeneity of Environmental Factors on Plant Community Structure in Temperate-Subtropical Ecological Transition Zone. Zhengzhou University. Dissertation of Master.

[134] Song, M. Z., Liu, H., Nie, H., et al. (2015). Resources of Rock Bryophytes Plant and Their Exploitation and Utilizations in Lushan Nature Reserve. Journal of Shanxi Agricultural University (Natural Science Edition). 35(06):566-570.

[135] Liu, Y., Tian, S., Pi, C. Y. (2015) Study on the Bryoflora in Nine Districts of Chongqing City[J]. Plant Science Journal. 33(2): 176-185. DOI: 10.11913/PSJ.2095-0837.2015.20176.

[136] Bakalin, V., Vilnet, A., & Xiong, Y. (2015). Mesoptychia chinensis Bakalin, Vilnet & Xiong sp. nov. (Jungermanniaceae, Marchantiophyta) and comments on the distribution of Mesoptychia south of the boreal zone in Asia. Journal of Bryology, 37(3), 192–201. https://doi.org/10.1179/1743282015Y.0000000009.

[137] Zhang, E. F., Gao, P., Zhao, J. C. (2014). Re - Survey on the Bryophyte of Lishan Nature Reserve. North Horticulture. (09):87-90.

[138] Tang, Y. X., Cao, T., Guo, S. L., et al. (2014). 6 New Genera Records of Bryophytes in Guangxi, China. Jour of Fujian Forestry Sci and Tech. 41(01):129-133. DOI: 10.13428/i.cnki.flk.2014.01.028.

[139] Tang, Y. X., Cao, T., Yu, J., Li, Q. (2013). New records of liverworts to Sichuan Province, China. Journal of Shanghai Normal University (Natural Science Edition). 42(02):182-185.

[140] Li, L., Ren, Z. J., Huang, Z. L., Guo, M. M., Zhao, Z. T. New records of Shandong bryophytes. Shandong Science. 26(01):28-34+43. DOI: 10.3976/j.issn.1002-4026.2013.01.007.

[141] Jia, Y., He, S. (2013). Species Catalogue of China Volume 1 Plants Bryophytes. Beijing: Science Press.

[142] Zhang, S. L., Zhang, X. J., Yong, S. P., et al. (2013). Wulanba Nature Reserve Comprehensive Study Collection. Beijing: China Forestry Press.

[143] Li, L. (2013). Study on the Liverworts of Shandong Province, China. Shandong Normal University. Dissertation of Master.

[144] He, Q. (2013). Spore output and cell surface structure of selected liverworts, and species diversity of Drepanolejeunea in China. East China Normal University. Dissertation of Master.

[145] He, L., Wu, A. Q., Zhang, R. B., et al. (2013). Survey on Species of Bryophytes on Rock in Sidonggou Scenic Area of Chishui. Hubei Agricullural Sciences. 52(20):4893-4897.

[146] Guo, M. M., Ren, Z. J., Li, L., et al. (2013). Effect of Environmental Changes on Liverworts in Shandong Province, China. Journal of Yantai University (Natural Science and Engineering Edition). 26(04):265-270.

[147] Tian, Y. L., Wang, W. H., Yan, T. Y., et al. (2013). Species Diversity of Floor Bryophytes in Different Vegetations in Baihua Mountain National Nature Reserve, Beijing, Chin. Bulletin of Botanical Research. 33(04):398-403. DOI: 10.7525/j.issn.1673-5102.2013.04.003.

[148] Li, S., Shen, L., Yu, J., et al. (2012). Newly recorded bryophyte genus and species from Jiangsu Province, China. Journal of Shanghai Normal University (Natural Sciences). 41(03):307-311.

[149] Wang, G. H. (2012). Study on the species diversity and flora of main mountain bryophytes in Shanxi Province. Beijing: Ocean Publishing House.

[150] Chen, H. T., et al. (2012). Fengyang Shanzhi. Beijing: China Forestry Press.

[151] Zhou, G. Y. (2012). Taxonomic Studies on *Chiloscyphus* - *Heteroscvphus* -*Lophocolea* Group in China. Hangzhou Normal University. Dissertation of Master.

[152] He, Z. X., Yan, Y. H., Ma, Q. X., et al. (2012). The bryophyte diversity of the Danxia landform in Hunan, China. Biodiversity Science. 20, 522-526. DOI: 10.3724/SP.J.1003.2012.05216.

[153] Rizwangul, E., Mamtimin, S., Wang, M. Z. (2012). New Distribution of Liverworts in Xinjiang. Xinjiang Agricultural Sciences. 49(04):708-715. DOI: 10. 6048/j. issn. 1001-4330. 2012. 04. 017.

[154] Shi, X. Q., Chen, J. W. (2012). Study on Bryophytes Flora in Tianma Nature Reserve of Anhui Province China. Journal of Anhui Normal University (Natural Science). 35(02):158-162. DOI: 10.14182/j.cnki.1001-2443.2012.02.022.

[155] Zhang, X. Q. (2011). Species Diversity of Liverworts and Hornworts of Daiyunshan Nature Reserve, Fujian, China. East China Normal University. Dissertation of Master.

[156] Sun, Y. (2011). Study on Species Diversity of Bryophytes in Jianfengling Nature Reserve. Hainan University. Dissertation of Master.

[157] Deng, J. J. (2011). Ecological diversity Report of the Epiphyllous Liverworts from Guizhou Province, China. Guizhou University. Dissertation of Master.

[158] Shen, L. (2011). Studies on diversity and ecology of bryophytes of the cities in North of Jiangsu Province, China. Shanghai Normal University. Dissertation of Master.

[159] Song, H. T. (2011). The diversity of Bryophytes in Zhenjiang, Changzhou and the comparison with its neigh boring areas in the Yangtze River Delta. Shanghai Normal University. Dissertation of Master.

[160] Wang, Y. Y. (2011). Effects of habitat fragmentation on bryophytes richness and genetic diversity in Thousand-Island Lake region, Zhejiang. East China Normal University. Dissertation of Master.

[161] Yan, X. L., Bao, W. K. (2011) Ground bryophyte diversity in secondary birch forests in western Sichuan, China. Biodiversity Science. 19, 327-334. DOI: 10.3724/SP.J.1003.2011.08166.

[162] Gao, Q., Wu, Y. H. (2010). Genera Hepaticopsida etAnthocerotopsida Sinicorum. Beijing: Science Press.

[163] Song, H. T., Guo, S. L., Shen, L., et al. (2010). Bryophyte Diversity and Relationship between Distribution and Environmental Factors in Bao - huashan Natural Reserve. Jiangsu Province Journal of Mountain Agriculture and Biology. 29(06):482-489. DOI: 10.15958/j.cnki.sdnyswxb.2010.06.002.

[164] Zhang, J. J. (2010). The Study on Ditrichaceae, Batramiaceae,and Lepidoziaceae in Guangdong and Hainan Province. Shanghai Normal University. Dissertation of Master.

[165] Liu, X. M., Guo, Y. R., Liu, R. L., et al. (2010). Jiangxi Qiyunshan Nature Reserve Comprehensive Scientific Expedition, Beijing: China Forestry Press.

[166] Wang, J. (2010). Taxonomic studies on Chinese Leieuneaceae. East China Normal University. Dissertation of Doctor.

[167] Yan, X. L. (2010). The Study On The Bryoflora And Its Classification Of Yangjifeng Nature Reserve. Zhejiang Forestry University. Dissertation of Master.

[168] Zhao, Y. (2010). The Study on Bryoflora Insaihanwula Nature Reserve. Inner Mongolia University. Dissertation of Master.

[169] Liu, B., Jiang, Y. F., Li, Q., et al. (2010). Epiphytic Bryophytes in Xiaoxi National Nature Reserve in Hunan. Life Science Research. 14(01):34-37. DOI: 10.16605/j.cnki.1007-7847.2010.01.005.

[170] Bai, X. L., Li, Z. G., Hu, T. H., et al. (2009). Bryophytes in Helan Mountain. Yinchuan: Ningxia People 's Press.

[171] Han, G. Y. (2009). Study on liverworts in Bailongjiang Valley in Gansu Province, Nw China. Shandong Normal University. Dissertation of Master.

[172] Cheng, X. F. (2009). Species Diversity and Distribution of Liverworts and Hornworts in Guangdong, China. East China Normal University. Dissertation of Master.

[173] Wang, J. (2009). Studies on flora and ecology of bryophytes in Suzhou and Yixing City Jiangsu Province, China. Shanghai Normal University. Dissertation of Master.

[174] Niu, Y. (2009). Studies on the Bryoflora and Bryum dill of Cuihua Mountain. Northwest University. Dissertation of Master.

[175] Wu, Y. H., Chen, L., Li, W., et al. Primary Study on Hepaticae from Gansu Province. Bulletin of Botanical Research. 29(05):607-614.

[176] Shi, X. Q., Wu, M. K., Zhang, X. P. (2009). Studies on liverwort F lora in Huangshan Mountain. Journal of Wuhan Botanical Research. 27(04):368-372.

[177] Zhang, E. F., Li, L., Zhao, J. C. (2009). A Preliminary Study of the Bryoflora of Pangquangou National Nature Reserve in Shanxi Province. Journal of Wuhan Botanical Research. 27(01):108-112.

[178] 王玲,郑荣周,何飞,等.四川西部地区苔藓植物区系研究[J].四川林业科技,2009,30(01):18-23.

[179] Gao, Q., Wu, Y.H. (2008). Flora Bryophytorum Sinicorum. Beijing: Science Press.

[180] Zhao, J. C., Wu, Y. F., Guan, W. L., et al. (2008). Laocha and Biodiversity Research in Tuoliang Nature Reserve, Hebei Province. Beijing: Science Press.

[181] Shi, R. P. (2008). Species Diversity and Distribution of Liverworts and Hornworts in Hainan Island, China. East China Normal University. Dissertation of Master.

[182] Shi, C. L. (2008). Studies on bryophyte of the Nabanhe National Nature Reserve, Yunnan Province. Shanghai Normal University. Dissertation of Master.

[183] Xu, C. Z. (2008). Studies on Species Diversity and Ecology of Epiphytic Bryophytes in Yangtze River Delta. Shanghai Normal University. Dissertation of Master.

[184] Zhang, E. F. (2008). The Study on Bryoflora of Pangquangou Nature Reserve in Shanxi Province. Hebei Normal University. Dissertation of Master.

[185] Zhang, Y. M. (2008). Study on Bryoflora and Ecology of Bryophytes in Sangong River Valley, Xinjiang. Graduate School of Chinese Academy of Sciences (Shenyang Institute of Applied Ecology). Dissertation of Doctor.

[186] Zhou, H. H. (2008). Urban Plant Diversity and Conservation Planning in Sanming County. Fujian Agriculture and Forestry University. Dissertation of Master.

[187] Li, Y. Y., Zhang, J. J., Cao, T. (2008). Biodiversity of bryophytes and their character isticson the aluval isands in the estuary of the Yangtze River. Journal of Shanghai Normal University (Natural Science Edition). (05):504-512.

[188] Wu, Y. H., Gao, Q., Cheng, G. D. (2008). Primary Study on Hepaticae from Mt. Qilian. Bulletin of Botanical Research. (02):147-150.

[189] Wang, W. H., Jia, Y., Yao, J., et al. (2008). Bryophytes’ Survival Status in Songshan Natural Reserve. Journal of Beijing Agricultural College. (01):54-60. DOI: 10.13473/j.cnki.issn.1002-3186.2008.01.002.

[190] Peng, T., Zhang, Z. H. (2007). New records of the bryophytes in Xiangzhigou Scenic Spot Guizhou Province. Journal of Guizhou Normal University (Natural Sciences) (02):20-24. DOI: 10.16614／j. cnki. issn1004-5570.2007.02.00.

[191] Sun, Y., Shao, X. M., Liu, X. C., et al. (2007). Bryophyte species diversity in main forest vegetations in Dongling Mountain of Beijing. Chinese Journal of Ecology. (11):1725-1731. DOI: 10.13292／j.1000－4890.2007.0313.

[192] Nan, Z. (2007). Hepatic flora of Maoershan Nature Reserve, Guangxiand synopsis of the hepatic flora of Guangxi. East China Normal University. Dissertation of Master.

[193] Yang, N. (2007). Study on Bryophyte Diversity and Flora of Ma Yang River Nature Reserve. Guizhou University. Dissertation of Master.

[194] Zhou, Y. (2007). Study on Flora of bryophytes in Legongshan Nature Reserve. Guizhou University. Dissertation of Master.

[195] Song, M. F. (2007). Study on Bryophytes in Taibai Mountain Nature Reserve. Northwest China University. Dissertation of Master.

[196] Ren, Y., Liu, M. S., Tian, L. H., et al. (2006). Research and Management of Biodiversity in Taibai Mountain Nature Reserve. Beijing: China Forestry Press.

[197] Wu, Y. F., Zhao, J. C., Cheng, J., et al. (2006). Scientific Investigation and Biodiversity Study of Maojingba Nature Reserve in Hebei Province. Beijing: Science Press.

[198] Wu, C. Z. (2006). Species Diversity and Flora of Bryophytes in Northwest Hunan, China. Guizhou University. Dissertation of Master.

[199] Shi, X. Q. (2006). Liverwort flora of Bawangling Nature Reserve and the correlations between the liverwort flora of Hainan Islandand adjacent islands. East China Normal University. Dissertation of Master.

[200] Liu, X. Z., et al. (2006). Scientific investigation and rare plant community research of Matoushan Nature Reserve in Jiangxi Province. Beijing: China Forestry Press.

[201] Li, F. X. (2006). Species and Ecosystem Diversity of Bryophyte in Foping Nature Reserve. East China Normal University. Dissertation of Master.

[202] Tian, G. Q. (2006). Study on the Bryophyte Flora and Ecology of Mountainous Hills and Adjacent Sandy Land in Northern Yanshan. Inner Mongolia University. Dissertation of Master.

[203] Zhang, Z. (2006). Studies on Species Diversity and Distribution Pattern of Bryophytes in Wuxi City, Jiangsu Province. Shanghai Normal University. Dissertation of Master.

[204] Yang, Z. P. (2006). Study on Bryophyte Diversity and Flora of S-W Hubei. Guizhou University. Dissertation of Master.

[205] Zhao, C. H. (2006). Bryoflora, ecology and travertine Deposition of bryophytes in Malinghe River Valley. Guizhou Normal University. Dissertation of Master.

[206] He, L. (2006). Study on Species Diversity of Bryophytes in the Southeast of Chongqing, China. University. Dissertation of Master.

[207] Wang, X. D. (2006). The Study on the classification and flora of bryophytes in Helan Mountain. Inner Mongolia University. Dissertation of Master.

[208] Li, X. N. (2006). Bryophytes of Karst Valleys in Luoping County, Yunnan Province. Guizhou Normal University. Dissertation of Master.

[209] Wang, Z. J. (2006). The Floristic Study of Higher Plant from Hebei Mountain. Hebei Normal University. Dissertation of Master.

[210] Peng, T. (2006). Diversity of the Bryophytes and Its Relationship with Travertine Deposition of Stream waterfalls in Xiangzhigou, Guizhou. Guizhou Normal University. Dissertation of Master.

[211] Xiang, J., Hu, Z. X., Fang, Y. P., et al. (2006). List of the Bryophytes on Daqishan Hill in Tuanfeng County，Hubei Province，China. Journal of Huanggang Normal University. (06):47-53+78.

[212] Wu, Y. H., Gao, Q. (2006). Notes on Chinese *Cyathodium* Kunze. Bulletin of Botanical Research. (05):522-526.

[213] Zhang, Z. H., Chen, J. K. (2006). Marchantiophyta and Anthocerophyta in Guizhou province, P. R. China. Journal of Bryology. 28(3), 170–176. DOI: 10.1179/174328206X120031.

[214] Huang, Y. X. (2005). The Preliminary Study on the Liverworts of Sichuan Province I-The Study on the Liverworts of Mt. Jinfo. Shandong Normal University. Dissertation of Master.

[215] Chen, Y. (2005). Studies on Distribution Patterns of Bryophytes and its Relationships with the Environments in Shanghai City, China. Shanghai Normal University. Dissertation of Master.

[216] Zhai, D. C., Yang, L. Q., Zhu, R. L. (2005). Study on Bryophytes in Evergreen Broadleaved Forests of Daweishan Nature Reserve, Yunnan Province, China. Journal of East China Normal University (Natural Science). (Z1):188-197.

[217] Zhai, D. C. (2004). Study on the Liverwort Flora and Ecological Distribution of Bryophytes in Evergreen Broadleaved Forests of Daweishan Nature Reserve. East China Normal University. Dissertation of Master.

[218] Wang, Y. (2004). The Study on the classification and flora of bryophytes in Qilaotu Mountain, Inner Mongolia. Inner Mongolia University. Dissertation of Master.

[219] Zhang, Z. H., Li, X. N., Peng, T., et al. (2004). New Records of Luminous Liverworts from the Karst Cavesof Guangxi Province, P. R. China: *Cyathodium* *Cavernarum* Kunze and *C. Smaragdium* Schiffin ex Keissler (Cyathodiaceae, Hepaticae). Casologica Sinica. (02):70-73.

[220] 曹同,高谦,孙军,等. (2004). *Scapania macroparaphyllia* a new species of Scapania (Scapaniaceae) from Xizang China. Acta Phytotaxonomica Sinica. (02):180-182.

[221] Gao, Q. (2003). Flora Bryophytorum Sinicorum. Beijing: Science Press.

[222] Yuan, Z. L. (2003). The Floristic Geography and Ecological Distribution of Bryophytes in Henan. Henan Agricultural University. Dissertation of Master.

[223] Huang, Y. D., Xie, Q. (2003). A Preliminary Study on the Ecological Distribution of Bryoflora Karst Stone Hill in Guilin. Casologica Sinica. (04):50-56.

[224] Zhang, Y. M., Lin, Q., Zhang, R. (2002). New records of bryophytes in Shandong. Journal of Shandong Normal University（Natural Science）. (04):81-85.

[225] Liu, X. Z., Xiao, Z. Y., Ma, J. H., et al. (2002). Scientific investigation and forest ecosystem research of Jiulianshan Nature Reserve in Jiangxi Province. Beijing: China Forestry Press.

[226] Zhao, J. C., Cui, Y. W., Ye, Y. Z., et al. (2002). An Analysis on the Flora and Geographical Elements of the Family Porellaceae in Liankang Mountain Nature Reserve Henan Province. Geography and Territorial Research. (04):107-110.

[227] Zhao, W. L., Liu, S. X., Huang, J., et al. (2002). A study on the bryophyta plants resources in Hubei，China -V A preliminary list of the mosses of Triangle Hill in Xishui County, Hubei Province, China. Journal of Huanggang Normal University. (06):39-45.

[228] Zhao, J. C., Cui, Y. W. (2002). Study on new recorded genera of Hepaticae in Hebei province Chian--- I. *Frullania* Raddi. Bulletin of Botanical Research. (04):412-416.

[229] Sun, J., Cao, T., Gao, Q. (2002) A Liverwort Species of Genus Horikawaella （Hepaticae）Is Found in China. Acta Botanica Yunnanica. (03):311-312. DOI: 0253-2700(2002)03-0311-02.

[230] Zhang, Y. M., Cao, T., Pan, B. R. (2002). Quantitative classification and ordination analysis on bryophytevegetation in bogda mountain Xinjiang. Acta Phytoecologica Sinica. (01):10-16.

[231] Jia, Y., Wu, P. C., Wang, M. Z. (2001). Bryoflora of mt. Wutong Shenzhen city South China. Guizhou Science. (04):16-22. DOI: 1003-6563(2001)04-0016-07.

[232] Liu, S. X., Peng, D., Qin, W., et al. (2001). Studies on the bryophyta plants resoucess in Hubei province－－ⅡThe bryophytes flora of Wuhan city. Journal of Central China Normal University (Natural Science Edition). (03):326-329. DOI: 10.19603/j.cnki.1000-1190.2001.03.022.

[233] Zhu, A. Q., Zhang, G. C., Xie, Q. (2000). The bryophytyes in the Yinzhulaoshan mountain of Gaungxi. Journal of Guangxi Normal University. (03):86-90. DOI: 10.16088/j.issn.1001-6600.2000.03.021.

[234] Liu, W. Q., Chu, Q. J., Liao, W. B., et al. (1999). Study on the bryophytes of Neilingding island nature reserve Guangdong province China. Guihaia. (4):303-307. DOI: 1000-3142(1999)04-0303-05.

[235] Ji, M. C., Liu, Z. L., Zhang, Z. Y., et al. (1999). A Preliminary Report on the Epiphyllous Liverworts Species from Jiangxi Province China. Jiangxi Science. (01):41-43. DOI: 1001-3679(1999)01-0039-0.

[236] Chen, R. R., Yang, S., Liu, N., et al. (1997). Preliminary studies on bryophytes of Qianling mountain. Journal of Guizhou Normal University (Natural Science). (01):48-59.

[237] Deng, X. (1994). A Preliminary study on moss species in Hunan province. Journal of Hunan Agricultural College. (02):132-137. DOI: 10.13331／j. cnki. Jhau.1994.02.007.

[238] Wang, W. H., Zhao, J. C. (1994). Study on the vertical distribution and community - types of bryophytes from the forest regions in Mt. Guancenshan Journal of Xinjiang University. (01):65-71.

[239] Furuki, T., Long, D. G. (1994). Aneura crateriformis, a new liverwort species from the East Himalaya and China. Journal of Bryology. 18(2), 281–286. https://doi.org/10.1179/jbr.1994.18.2.281

[240] Ji, M. C. Studies on the Bryophyta in Yunju Mountainin North Jiangxi Province. Acta Agriculturae Universitatis Jiangxicnsis. 1993(02):174-181. DOI: 10.13836／j. jjau.199302.

[241] Zhao, J. C. (1993). Study on the Bryophytes on Mt. Bogda, Xinjiang. Journal of Xinjiang University. (01):73-92.

[242] Kang, X. G., Zhang, S. M. (1992). A Taxonomic Catalogue of Bryophytas from Zhengfen Mountain. Journal of Jilin Agricultural University. (04):24-29+33-114. DOI: 10.13327／j. jjlau.1992.04.007.

[243] Zhu, R. L., Zhang, G. Z., Mao, X. R. (1992). Resources of epiphyllous liverworts in Baishanzu Nature Reserve of Zhejiang Province. Journal of Plant Resources and Enronment. (03):19-23.

[244] Li, C. L., Ji, M. C. (1991). A Preliminary Study of Bryophytes in Xishanmeiling Region. Acta Agriculturae Universitatis Jiangxicnsis. (01):47-50. DOI: 10.13836／j. jjau.1991009.

[245] Chang, H. X. (1989). Bryophytes in Lushan. Journal of Jiangxi University (Science Edition). (04):80-89.

[246] Chang, H. X. (1989). A Survey on the Bryophytes from the Jinggang Mountain in Jiangxi. Journal of Jiangxi University (Science Edition). (01):62-71.

[247] Ji, J. X., Chen, H. D. (1986). Study on bryophytes in Wuling Mountain of Hebei Province. Journal of Beijing Normal University (Natural Science Edition). (01):49-59. DOI: 10.19789／j.1004-9398.1986.

[248] Kang, X. G. (1985). A Hepaticaespecies is Newly Recorded in China – Frullania Oakesiana. Jilin Agricultural University. (04):18-19+103. DOI: 10.13327／j.jjlau.1985.04.00.

[249] Hattori S, Lin P J. (1985). A preliminary study of Chinese Frullania florae. J. Hat. Bot. Lab. 59: 123-169.

[250] Hu, R. L. (1958). Preliminary Investigation Report on Bryophytes in Dongting West Mountain of Taihu Lake. Journal of East China Normal University (Nature Science). (00):63-72.

# Supplementary Figures and Tables

## Supplementary Figures

**Supplementary Figure S1**. Maps of Provinces in China used in this study. (GS (2024) 0650)


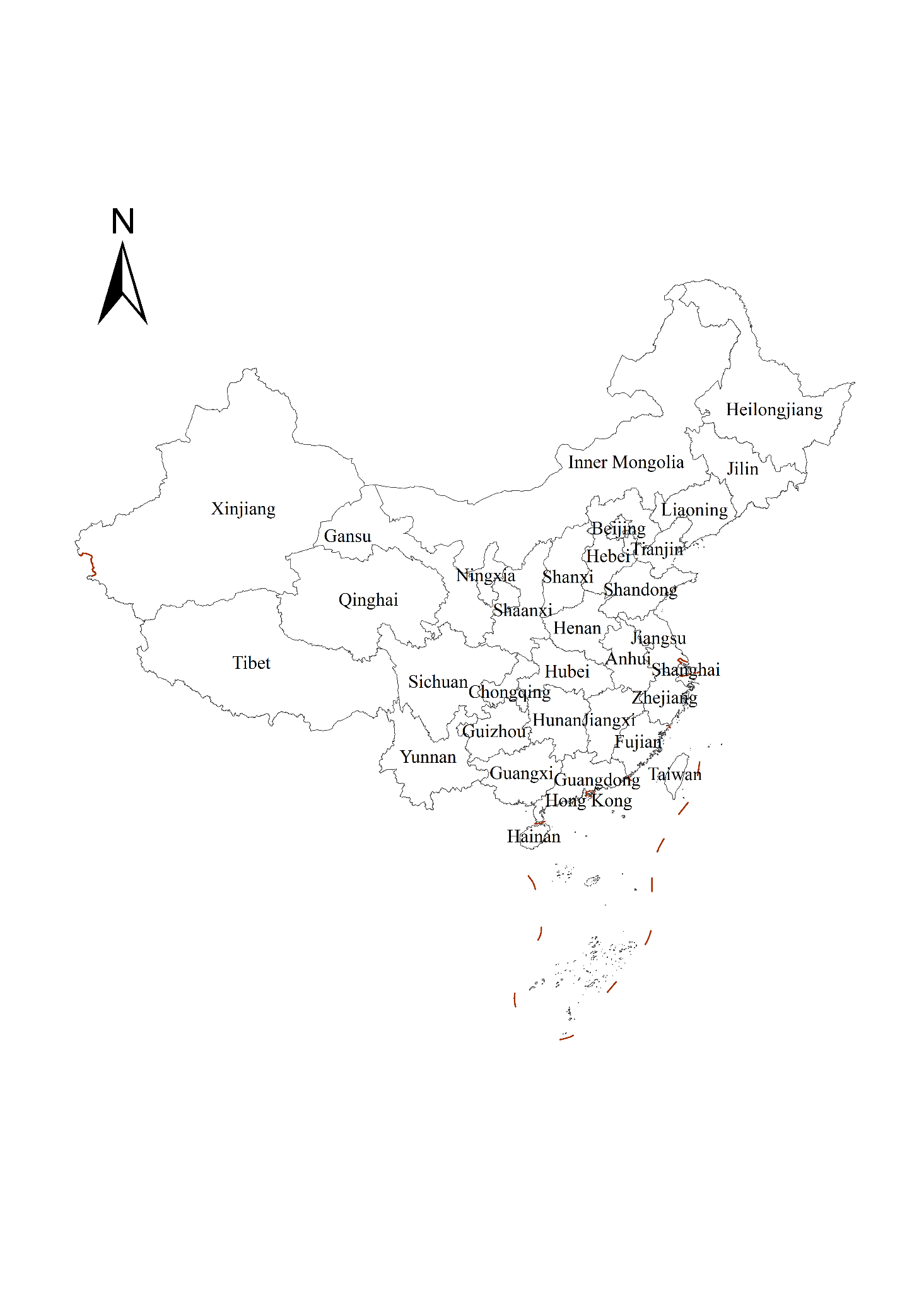


## Supplementary Tables

**Supplementary Table S1.** The species number of liverworts in each province in China from this study and two previous studies. Noted in the study of Jia & He (2013), Qian et al. (2016) and Song et al. (2021).

| Province | Jia and He  (2013) | Qian et al.  (2016) | Song et al.  (2021) | CoLChina  (2024) | This study |
| --- | --- | --- | --- | --- | --- |
| Anhui | 108 | 176 | 109 | 119 | 304 |
| Macau | - | - | - | 35 | 35 |
| Beijing | - | - | - | 18 | 46 |
| Fujian | 343 | 367 | 360 | 364 | 430 |
| Gansu | 82 | 76 | 103 | 101 | 233 |
| Guangdong | 211 | 244 | 215 | 152 | 314 |
| Guangxi | 241 | 270 | 297 | 269 | 506 |
| Guizhou | 291 | 360 | 369 | 311 | 644 |
| Hainan | 236 | 253 | 233 | 245 | 414 |
| Hebei | 68 | 64 | 69 | 58 | 91 |
| Henan | 13 | 69 | 31 | 18 | 140 |
| Heilongjiang | 149 | 148 | 153 | 161 | 184 |
| Hubei | 109 | 133 | 170 | 124 | 301 |
| Hunan | 200 | 225 | 215 | 224 | 266 |
| Jilin | 154 | 168 | 162 | 163 | 191 |
| Jiangsu | 36 | 61 | 50 | 45 | 74 |
| Jiangxi | 174 | 248 | 198 | 191 | 343 |
| Liaoning | 83 | 118 | 93 | 90 | 160 |
| Inner Mongolia | 91 | 109 | 94 | 102 | 133 |
| Ningxia | 14 | 16 | 14 | 16 | 18 |
| Qinghai | 16 | 15 | 16 | 20 | 44 |
| Shandong | 89 | 92 | 101 | 103 | 130 |
| Shanxi | 20 | 34 | 29 | 24 | 44 |
| Shaanxi | 104 | 168 | 214 | 118 | 312 |
| Shanghai | - | - | - | 45 | 53 |
| Sichuan | 388 | 390 | 357 | 392 | 514 |
| Taiwan | 483 | 519 | 482 | 494 | 526 |
| Tianjin | - | - | - | 10 | 10 |
| Xizang | 294 | 306 | 295 | 322 | 440 |
| Hong Kong | - | - | - | 165 | 170 |
| Xinjiang | 57 | 62 | 58 | 65 | 105 |
| Yunnan | 610 | 619 | 621 | 652 | 814 |
| Zhejiang | 254 | 279 | 267 | 270 | 445 |
| Chongqing | - | - | 171 | 151 | 319 |

Jia & He (2013), Qian et al. (2016) and Song et al. (2021): They combined each municipality except Chongqing which has a very large area with one of its neighboring provinces. Specifically, It combined Beijing and Tianjin with Hebei Province, Shanghai with Zhejiang Province, and Hong Kong and Macau with Guangdong Province. On the final map, China was divided into 29 province-level geographic units (“province” hereafter).
